# From therapeutic pathways to patient archetypes: a latent class analysis framework for Value-Based Healthcare benchmarking

**DOI:** 10.3389/frhs.2026.1789729

**Published:** 2026-10-21

**Authors:** Maialen Otamendi, Itxaso Alayo, Ania Gorostiza, Jon Cacicedo, Ane Fullaondo, Borja García-Lorenzo

**Affiliations:** 1 Group of Environmental Epidemiology and Child Development, Biogipuzkoa Health Research Institute, Donostia/San Sebastian, Spain; 2 Biosistemak Institute for Health System Research, Bilbao, Basque Country, Spain; 3 Red de Investigacion en Cronicidad, Atencion Primaria y Promocion de la Salud RICAPPS-(RICORS), Instituto de Salud Carlos III (ISCIII), Madrid, Spain; 4 Department of Radiation Oncology, Cruces University Hospital/Biobizkaia Health Research Institute, Barakaldo, Bizkaia, Spain

**Keywords:** value-based healthcare, latent class analysis, patient archetypes, benchmarking, breast cancer

## Abstract

**Background:**

Value-Based Healthcare (VBHC) focuses on Patient-Centred Outcomes (PCOs) to maximize healthcare value. Benchmarking across providers requires comparing outcomes among clinically and therapeutically comparable patients. However, traditional classification is based on biological features without accounting for the therapeutic trajectory. Establishing patient archetypes based on therapeutic pathways is therefore essential to prevent case-mix bias in VBHC benchmarking. The aim of this paper is to develop a model-based framework using Latent Class Analysis (LCA) to (i) identify patient archetypes in breast cancer and (ii) establish a probabilistic tool to classify new patients.

**Methods:**

A cohort of 2485 patients diagnosed with breast cancer between 2018 and 2020 was followed-up. Demographic and treatment variables were collected at baseline and at 6-months follow-up. LCA was applied to derive archetypes in a training set (80%). Fit and classification quality were assessed using aBIC, entropy, and posterior probabilities. Tool classification robustness was evaluated using Random Forest (RF) in a test set (20%), and generalizability was tested in an external multicenter cohort (*N* = 352).

**Results:**

A six-class solution (Arch-1—Arch-6) was selected, achieving optimal fit, parsimony, high entropy (0.932), and clinical interpretability. Evaluated against RF, the tool demonstrated high classification concordance (99.68% accuracy). In the external cohort, high individual classification certainty was maintained (mean posterior probability = 0.965), supporting consistent assignment across care settings.

**Conclusions:**

LCA provides a transparent, efficient and robust tool to identify patient archetypes and assign new patients based on therapeutic pathways, enabling fair benchmarking in VBHC. Prospective validation in broader cohorts is recommended to confirm transferability to other clinical conditions.

## Introduction

Value Based Healthcare (VBHC) proposes a management approach focused on Patient-Centred Outcomes (PCOs) prioritizing what matters most to patients, suggesting the linkage of PCOs to costs to determine the value of healthcare, hence estimating the health outcomes achieved per monetary unit invested (1–3). The VBHC approach seeks to achieve high value for patients, improving the healthcare delivery process and the economic sustainability of the healthcare system (1, 4). Even though the 5-year survival rate has considerably increased at 90% in breast cancer over recent years (5), a significant decrease in the quality of life (6) remains, together with high healthcare resource utilisation (7). Particularly, the increase of the survival rate has been yielded across a wide variety of multimodal therapeutic approaches (8) which plays a key role in the patient's quality of life (2, 9). These facts highlight the growing importance of VBHC in the breast cancer healthcare.

Within the VBHC framework, benchmarking requires comparing outcomes across providers for patients who are clinically and therapeutically comparable. Previous research has shown that therapeutic pathways strongly influence patient-reported outcomes, with distinct functional and symptomatic trajectories depending on the type of surgery, radiotherapy, chemotherapy or reconstruction received Gorostiza et al. (10). If benchmarking is conducted without accounting for these differences, performance comparisons may be confounded by case-mix rather than reflecting true differences in quality of care. Patient archetypes provide a structured way to group individuals with similar therapeutic pathways and clinical profiles, enabling fair, interpretable and actionable benchmarking. In the absence of such classification, benchmarking exercises risk generating biased conclusions, masking unwarranted clinical variation and limiting their usefulness for improving care delivery.

In this context, although patient classifications have been usually based on biological and molecular subtypes determining the therapeutic decisions, the literature has raised that this is not always the case due to unwarranted clinical variability (11–14). For this reason, VBHC have suggested that archetypes should be based on the patient's characteristics and therapeutic pathway (15, 16). Accounting for this heterogeneity is important for meaningful value comparisons and benchmarking across providers (17). Archetypes have been shown to have a significant impact on the PCO, the cornerstone of VBHC (10). For instance, it has been previously published that patients showing worse breast satisfaction results at 6 months with respect to baseline are those belonging to the archetypes that have undergone mastectomy (non-breast-conserving surgery). Likewise, it should be noted that archetypes including conservative surgery followed by radiotherapy without adjuvant chemotherapy showed the least differences at 6-month of follow-up, in both functional aspects and symptomatic burden, when comparing to the reference group. Then, grouping patients might help to identify response patterns and determine the most efficient therapeutic strategies including patients perspective (10, 15, 16).

VBHC benchmarking across healthcare centres has been shown to be helpful to identify lessons that may be learned from the best performers (15). Although patients are diagnosed with the same disease, their individual profiles might vary, and therefore affect the benchmarking accuracy across healthcare centres. To show a rigorous benchmarking, the VBHC suggests to define patient archetypes to ensure outcomes comparability across sites (16). A patient archetype is defined as a patient profile grouping patients with similar characteristics.

Previous research addressing the design of patient archetypes following a VBHC approach conducted a mixed approach combining clinical criteria and a Hierarchical Clustering on Principal Components (HCPC) technique (10, 16). However, HCPC requires a subjective decision regarding the number of groups and does not provide a probabilistic framework for allocating new patients. In contrast, the present study introduces Latent Class Analysis (LCA) as a model-based alternative that determines the optimal number of classes using statistical fit indices and yields posterior probabilities that enable automatic and uncertainty-aware classification. To our knowledge, this represents the first application of LCA to derive therapeutic-pathway-based archetypes in breast cancer within a VBHC framework. Therefore, this research has a twofold objective: first, to identify patient archetypes through an unsupervised, model-based approach using Latent Class Analysis (LCA); and second, to develop a probabilistic classification tool that assigns new patients to the previously defined archetypes based on posterior membership probabilities. These two aims are related but analytically distinct: the former concerns the discovery of the latent structure of therapeutic pathways, whereas the latter enables its operational use for benchmarking within the VBHC framework.

## Methods

### Study design

Population data of the *Organización Sanitaria Integrada Ezkerraldea-Enkarterri-Cruces* belonging to the Basque Health System-Osakidetza (Spain) was collected with the approval of the Basque Country Ethics Committee (PI2018107). The study population consisted of patients diagnosed with early breast cancer over 2018–2020 meeting the following eligibility criteria: i) aged over 18 years; ii) female; iii) newly diagnosed invasive breast cancer (stage I-III) or ductal carcinoma *in situ* (DCIS), and iv) receiving any treatment (surgery, radiotherapy, chemotherapy, hormone therapy and/or targeted therapy). Criteria for exclusion included patients with i) rare tumours, ii) lobular carcinoma *in situ*, or iii) metastatic disease.

### Data

Socio-demographic variables, Clinical-Related Outcomes (CROs) and Care-Process Related-Outcomes (CAPROs) were collected at baseline and followed-up at 6-months according to the standard set of variables provided by the International Consortium for Health Outcomes Measurements (ICHOM)^9,11^. These variables were collected for descriptive and benchmarking purposes; however, only demographic and treatment-related variables were used for the definition of patient archetypes.

### Empirical strategy

After conducting a descriptive analysis followed by statistical tests of the demographic, tumour, and treatment characteristics of the sample, the empirical strategy consisted of three steps: i) to identify the patient archetypes, ii) to design an automatic classification tool to allocate new patients to the previously identified archetypes, and iii) to evaluate the robustness of the classification tool using an alternative machine learning method. Patients with ductal carcinoma *in situ* (DCIS) were defined *a priori* as a separate reference archetype (Arch-0) based on clinical expert consensus and the non-invasive nature of the disease. Because DCIS follows a substantially simpler therapeutic pathway with a lower treatment burden compared to invasive breast cancer, pre-classifying these patients separately ensured that the subsequent LCA focused exclusively on capturing therapeutic pathway heterogeneity among clinically comparable invasive profiles, thereby preserving both the statistical stability and clinical interpretability of the derived archetypes. Then, sample was randomly split into two subsamples: the train dataset (80%) intended to identify the patient archetypes and to train the patient identification tool, and the test dataset (20%), to allocate new patients into the defined archetypes. External validation was also performed using a sample from the VOICE (Value-Based Healthcare for Outcomes in Breast and Lung Cancer in Europe) community (10, 16).

### Patient archetype definition

Latent Class Analysis (LCA) (18) was applied to identify the archetypes of patient diagnosed with breast cancer. LCA allows statistical inferences to determine the most appropriate number of groups. Then, patient archetypes were identified designing a Latent Class Model (LCM) where a latent categorical variable defined as patient archetype might explain the association among a set of observed variables. For the definition of archetypes, clinicians selected variables that capture the therapeutic pathway experienced by each patient. Accordingly, the LCA included demographic and treatment-related variables (age, type of breast surgery, type of axillary surgery, chemotherapy, radiotherapy and hormonotherapy). These variables were chosen because they reflect the sequence and intensity of treatments that shape patients' clinical trajectories and are known to influence patient-centred outcomes. Patient-centred outcomes were intentionally excluded from the classification process to avoid circularity, as they are later used as benchmarking outcomes within the VBHC framework. Patients with invasive ductal carcinoma (IDC), invasive lobular carcinoma (ILC) and other carcinoma (OC) were included in the LCA and classified according to the latent structure derived from the selected demographic and treatment-related variables.

The LCM parameters were estimated using the train cohort. For a given manifest variable $(Y_{j}),$ the probability of observing a particular response $y_{\;j\;}$ depends on the individual's membership in a specific latent class c. This conditional probability, denoted as $P(Y_{j}=y_{j}|L=c)$, allows for interpreting the profiles of the observed variables within each class. The probability of belonging to a latent class c, noted as P(L=c), defines the proportion of individuals allocated to that subgroup. By combining these probabilities under the assumption of local independence, where the manifest variables are independent within each latent class, the overall likelihood of observing the response pattern across all variables can be modelled as follows:

$$P(Y=y)=\overset{C}{\underset{c=1}{\sum }}\;P(L=c)\overset{M}{\underset{\;j=1}{\prod }}\;P(Y_{j}=y_{j}|L=c),$$

Where Y represents the set of manifest variables and y the observed responses. The model calculates the probability of observing a complete response pattern as the sum of probabilities across all latent classes. The assumption of local independence was assessed by examining bivariate residuals (BVRs) for all pairs of observed variables in the latent class model.

LCM were fitted using the train cohort, testing models with different number of classes, from one to eight, in order to identify the optimal number of archetypes. Akaike Information Criterion (AIC), Bayesian Information Criterion (BIC), the sample-size adjusted BIC (aBIC) and goodness-of-fit statistics (*χ*^2^ and G^2^) were considered to assess model fit. Classification quality was further assessed using entropy and average posterior probabilities of class membership. These indicators were used to select the model with the most appropriate balance between fit, parsimony and classification quality. Patient classification tool and robustness evaluation.

The application of LCA also enables the classification of patients into archetypes. Once the archetypes were identified in the train cohort, patients in the test dataset were allocated into their most likely subgroup. The LCM, using the test dataset, enabled the prediction of the latent variable—archetype—for the test sample by estimating the probability of each patient to belong to all archetypes based on on the selected demographic and treatment-related variables. Once these probabilities were calculated for all archetypes, the new patient was classified within the most probable latent class.

To evaluate the classification consistency and robustness of the proposed tool, a Random Forest (RF) supervised machine learning classifier (18) was trained using the LCA-assigned archetypes as the outcome variable. The RF classifier was used to develop a model in the train subsample (80%). K-fold cross-validation was applied (*k* = 5). Model with the best parameters was applied to the test dataset 20%, classifying the patients into archetypes. The classifications obtained with the RF model were compared with those derived from the LCA to confirm the consistency of the archetypes identified by the LCA for the same test sample, using a confusion matrix, along with metrics such as sensitivity, specificity and accuracy. It should be noted that, once the archetypes were defined with LCA, the RF model was used to test the robustness of the classification of new patients into those pre-established archetypes, rather than to validate the latent structure itself.

To evaluate the generalizability and robustness of the patient classification derived from LCA, an external validation was performed using an independent multicenter dataset from the VOICE community (*N* = 352) (10, 16). The LCA parameters estimated on the derivation cohort were applied to calculate posterior membership probabilities for each patient in the external cohort, assigning them to their most probable archetype. The consistency of the archetypes across cohorts was evaluated using two complementary approaches: individual classification certainty, assessed via the average posterior probability (APP) of class membership for patients in the external cohort, and a descriptive comparison of the distribution of classification variables within each archetype between cohorts.

Analyses were performed using R version 4.2.2.

## Results

### Description of the study population

The cohort was composed of 2,485 patients. Missing data by classification variable are reported in Table 1. Patients' mean age at diagnosis was 60.91 years (SD = 13.15) and 54.4% had invasive ductal carcinoma. After excluding patients with missing data and separating patients with ductal carcinoma *in situ* (*n* = 231), who were assigned directly to Arch-0 based on clinical criteria, the analytic sample comprised 1,570 patients, split into a training set (*n* = 1,256, 80%) used to identify the archetypes, and a test set (*n* = 314, 20%) used to evalute the classification of new patients. The flow chart in Figure 1 provides a more detailed overview of the analytical steps.

**Table 1:** Patient characteristics^a^.

Variables	Sample size *N* = 2,485
Patient characteristics	
Age at diagnosis	
(0, 50)	515 (20.72%)
(50, 70)	1,106 (44.51%)
(70, 97)	477 (19.20%)
NA (missing)	387 (15.57%)
Tumour characteristics	
Tumour type	
Ductal carcinoma in situ	279 (11.23%)
Invasive ductal carcinoma	1,351 (54.37%)
Invasive lobular carcinoma	174 (7.00%)
Other carcinoma	92 (3.70%)
NA (missing)	589 (23.70%)
Treatment characteristics	
Surgery	
Breast conserving surgery	1,440 (57.95%)
MWR	357 (14.37%)
MWoR	188 (7.57%)
NA (missing)	500 (20.12%)
Surgery to axilla	
None	747 (30.06%)
Sentinel lymph node biopsy	1,407 (56.62%)
Axillary clearance	321 (12.92%)
Axillary sampling	8 (0.32%)
NA (missing)	2 (0.08%)
Chemotherapy	
No	2,139 (86.08%)
Yes	346 (13.92%)
Radiotherapy	
No	1,474 (59.32%)
Yes	1,011 (40.68%)
Hormonal therapy	
No	1,504 (60.52%)
Yes	981 (39.48%)
Targeted therapy	
No	2,383 (95.90%)
Yes	102 (4.10%)

a Percentages are calculated over the total cohort (*N* = 2,485), including missing values.

MWR, Mastectomy with immediate reconstruction; MWoR, Mastectomy without immeadiate reconstruction.

**Figure 1:**
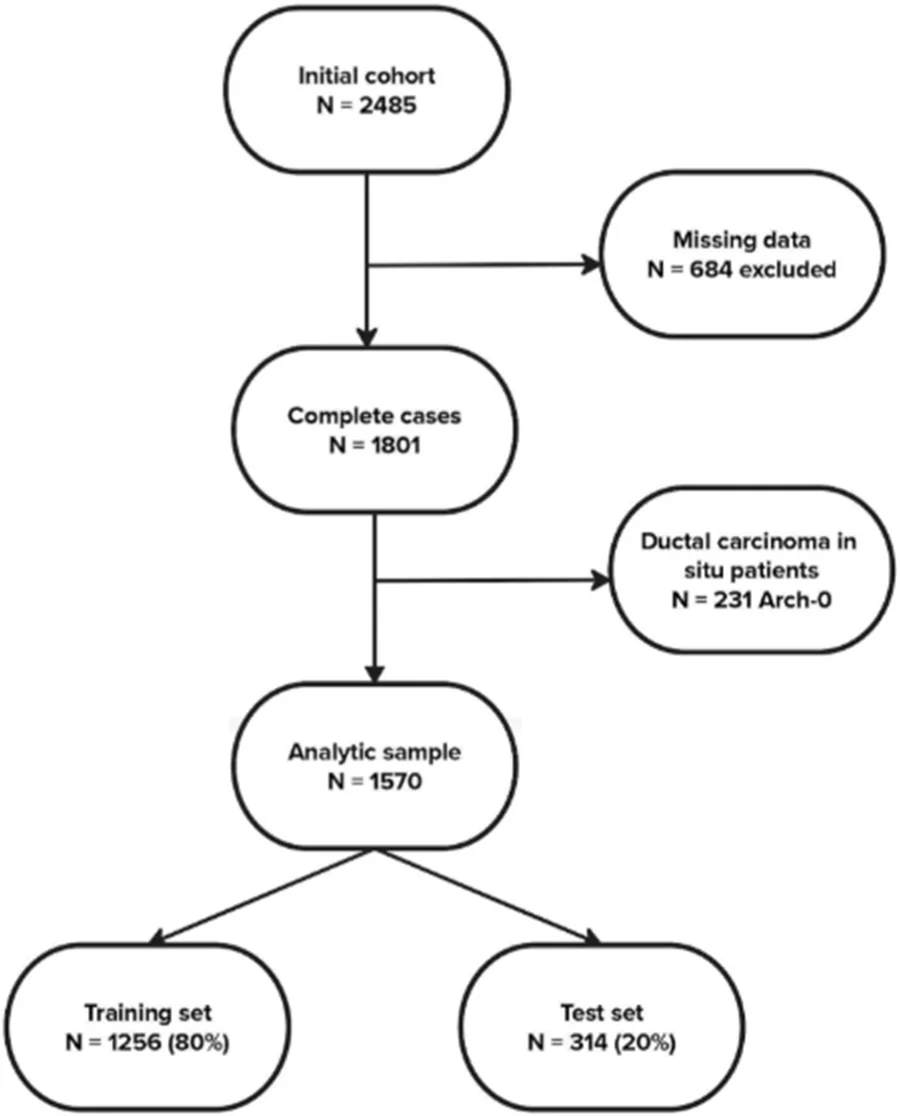
Flow-chart patient selection and analytic sample derivation. Arch-0, Archetype 0.

### Patient archetype definition

The parameters of model fit for the eight models, that give rise to one to eight latent classes—defined as patient archetypes—respectively, are shown in Table 2. Although the unadjusted BIC was lowest for the five-class model, the sample-size adjusted BIC (aBIC), which applies a less stringent penalty for model complexity in larger samples, was minimised for the six-class model (aBIC = 9735.97), below both the five- and seven-class solutions. The AIC continued to decrease up to the eight-class model, but this improvement in fit was accompanied by a clear deterioration in classification quality: entropy fell from 0.932 in M6 to 0.848 in M8, the minimum average posterior probability dropped from 0.92 to 0.71. In contrast, M6 combined adequate G^2^ and *χ*^2^ statistics with the highest entropy and posterior probabilities among the eight models. Beyond these statistical criteria, the six archetypes identified under M6 showed clear clinical interpretability and were consistent with the six patient archetypes previously described under HCPC within the VOICE consortium, which had originally motivated this study. Considering jointly model fit, classification quality, parsimony and clinical concordance, the six-class model (M6) was retained as the most appropriate solution. The assumption of local independence was also assessed in the selected six-class model by examining bivariate residuals (BVRs) for all pairs of observed variables. The highest BVR value was 1.97, observed for the association between age and axillary surgery, and no substantial violations of local independence were identified. Table 3 presents the most representative patient for each archetype, while Table 4 provides a descriptive analysis of the data across all six archetypes.

**Table 2:** Comparison of LCA models.

Models	AIC	BIC	aBIC	G²	*χ*²	Entropy	Mean APP	Class sizes, N (%)
M1	10,769.58	10,820.94	10,789.17	1,470.16	2,556.27	1.00	1	1,256 (100%)
M2	10,197.14	10,304.99	10,238.28	875.72	2,105.17	0.804	0.96; 0.95	648 (51.6%); 608 (48.4%)
M3	9,934.42	10,098.76	9,997.11	591.00	1,174.70	0.804	0.79; 0.96; 0.89	156 (12.4%); 635 (50.6%); 465 (37.0%)
M4	9,756.99	9,977.83	9,841.24	391.57	420.93	0.815	0.93; 0.92; 0.95; 0.79	486 (38.7%); 479 (38.1%); 129 (10.3%); 162 (12.9%)
M5	9,651.13	9,928.46	9,756.93	263.71	267.17	0.872	0.93; 0.98; 0.96; 0.83; 0.91	143 (11.4%); 120 (9.6%); 310 (24.7%); 127 (10.1%); 556 (44.3%)
M6	9,608.62	9,942.44	9,735.97	199.20	207.46	0.932	0.97; 0.98; 0.95; 0.93; 0.97; 0.92	421 (33.5%); 121 (9.6%); 144 (11.5%); 40 (3.2%); 368 (29.3%); 162 (12.9%)
M7	9,593.39	9,983.70	9,742.29	161.97	153.63	0.916	0.95; 0.94; 0.87; 0.99; 0.95; 0.84; 0.96	144 (11.5%); 40 (3.2%); 152 (12.1%); 109 (8.7%); 416 (33.1%); 21 (1.7%); 374 (29.8%)
M8	9,584.47	10,031.28	9,754.93	131.05	124.34	0.848	0.9; 0.93; 0.9; 0.85; 0.88; 0.83; 0.96; 0.71	82 (6.5%); 43 (3.4%); 415 (33.0%); 63 (5.0%); 73 (5.8%); 292 (23.2%); 151 (12.0%); 137 (10.9%)

AIC, Akaike Information Criterion; BIC, Bayesian information Criterion; G^2^, likelihood ratio chi-square.

χ^2^, The Pearson χ² goodness-of-fit statistic.

aBIC: sample-size adjusted Bayesian Information Criterion (Sclove, 1987).

Entropy: relative entropy R^2^ (Ramaswamy et al., 1993); values closer to 1 indicate better class separation.

Mean APP (range): range of average posterior probabilities of class membership across classes.

**Table 3:** Most representative patient per archetype.

Archetypes (*N* = 1,487)^a^	Age at diagnosis	Surgery	Surgery to axilla	Chemotherapy	Radiotherapy	Hormonotherapy
Arch-0, *N* = 231	(50–70)	Breast conserving surgery	None	No	Yes	No
Arch-1, *N* = 144	<50	MWR	Sentinel lymph node biopsy	No	No	No
Arch-2, *N* = 121	>70	MWoR	Axillary clearance	No	No	No
Arch-3, *N* = 421	(50–70)	Breast conserving surgery	Sentinel lymph node biopsy	No	Yes	Yes
Arch-4, *N* = 40	<50	MWR	Axillary clearance	Yes	Yes	Yes
Arch-5, *N* = 368	(50–70)	Breast conserving surgery	Sentinel lymph node biopsy	No	No	No
Arch-6, *N* = 162	(50–70)	Breast conserving surgery	Sentinel lymph node biopsy	Yes	Yes	Yes

a Derivation cohort: 80% of the cohort (*N* = 1,259) + arch-0 cohort (*N* = 231).

MWR, Mastectomy with immediate reconstruction; MWoR, Mastectomy without immediate reconstruction.

The representative patient for each archetype corresponds to the modal category of each classification variable within that archetype.

**Table 4:** Description of archetypes under LCA.

Variables	Arch-0^a^ (*N* = 231)	Arch-1 (*N* = 144)	Arch-2 (*N* = 121)	Arch-3 (*N* = 421)	Arch-4 (*N* = 40)	Arch-5 (*N* = 368)	Arch-6 (*N* = 162)
Age at diagnosis:							
<50	50 (21.65%)	81 (56.25%)	10 (8.26%)	70 (16.63%)	32 (80%)	68 (18.48%)	66 (40.74%)
[50,70]	153 (66.23%)	63 (43.75%)	40 (33.06%)	243 (57.72%)	8 (20%)	215 (58.42%)	85 (52.47%)
>70	28 (12.12%)	0 (0%)	71 (58.68%)	108 (25.65%)	0 (0%)	85 (23.10%)	11 (6.79%)
Surgery:							
Breast conserving surgery	184 (79.65%)	0 (0%)	0 (0%)	420 (99.76%)	0 (0%)	365 (99.18%)	162 (100%)
MWoR	13 (5.63%)	0 (0%)	121 (100%)	1 (0.24%)	7 (17.5%)	0 (0%)	0 (0%)
MWR	34 (14.72%)	144 (100%)	0 (0%)	0 (0%)	33 (82.5%)	3 (0.82%)	0 (0%)
Surgery to axilla:							
None	154 (66.67%)	1 (0.69%)	7 (5.79%)	14 (3.33%)	0 (0%)	30 (8.15%)	0 (0%)
Sentinel lymph node biopsy	75 (32.47%)	114 (79.17%)	44 (36.36%)	390 (92.64%)	3 (7.5%)	296 (80.43%)	125 (77.16%)
Axillary clearance	1 (0.43%)	27 (18.75%)	67 (55.37%)	17 (4.04%)	37 (92.50%)	42 (11.41%)	37 (22.84%)
Axillary sampling	1 (0.43%)	2 (1.39%)	3 (2.48%)	0 (0%)	0 (0%)	0 (0%)	0 (0%)
Chemotherapy	1 (0.43%)	19 (13.19%)	15 (12.40%)	0 (0%)	40 (100%)	1 (0.27%)	162 (100%)
Radiotherapy	120 (51.95%)	16 (11.11%)	27 (22.31%)	350 (83.14%)	37 (92.5%)	65 (17.66%)	137 (84.57%)
Hormonotherapy	97 (41.99%)	57 (39.58%)	25 (20.66%)	421 (100%)	35 (87.5%)	0 (0%)	110 (67.90%)

a Arch-0 was defined *ex-ante* the estimation of the Latent Class Models following the clinical expert's criteria.

MWR, Mastectomy with immediate reconstruction; MWoR, Mastectomy without immediate reconstruction.

The six archetypes identified by LCA showed different treatment patterns. Arch-3 (*n* = 421) and Arch-6 (*n* = 162) were the two most frequent breast-conserving treatment pathways. Both included breast-conserving surgery, sentinel node biopsy, radiotherapy and hormonal therapy, but they differed mainly in the use of chemotherapy. While almost no patients in Arch-3 received chemotherapy, all patients in Arch-6 had this treatment, suggesting a more aggressive tumour profile. Arch-5 (*n* = 368) also included mainly breast-conserving surgery, but with less use of radiotherapy and no hormonal and chemotherapy, which may indicate a different tumour profile. Arch-1 (*n* = 144) and Arch-2 (*n* = 121) were mainly based on mastectomy, but showed clear differences in age and treatment. Arch-1 included mainly younger patients, and mastectomy was usually followed by immediate reconstruction. In contrast, Arch-2 included mostly older patients and mastectomy was generally performed without reconstruction and with more extensive axillary surgery. Arch-4 (*n* = 40) was the smallest archetype and included mostly young patients (80% aged under 50 years). This group received the most intensive treatment, combining mastectomy and axillary clearance with chemotherapy, radiotherapy and hormonal therapy in all patients. Finally, Arch-0 (*n* = 231), defined *a priori* as patients with ductal carcinoma *in situ*, was characterised mainly by breast conserving surgery without axillary intervention and very limited use of chemotherapy, in line with the non-invasive nature of this condition.

### Patient classification tool and robustness evaluation

The classification tool evaluation using LCA showed a high overall accuracy (99.68%) across the 5-fold cross-validation using RF, providing stability and robustness of the LCA. Performance metrics by archetype revealed good classification for most archetypes, with sensitivities and specificities of 100% for Arch-2, Arch-3, Arch-5 and Arch-6, and slightly lower values for Arch-1 and Arch-4. These metrics were derived from the confusion matrix (Supplementary Table S2), which shows the classification counts for each archetype. Supplementary Table S3 shows the performance metrics from the 5-fold cross validation between the LCA and the RF.

To assess the generalizability of the latent class structure, a descriptive comparison of the distribution of classification variables across archetypes was performed between the original and the external VOICE cohort (see Supplementary Table S4). Although the overall pattern of variable distribution within each archetype was similar across the two cohorts, some differences were observed, most notably in the proportions of age, surgical type and treatment-related variables, likely reflecting differences in case-mix and clinical practice between centres rather than a failure of the archetype structure itself. This concordance is interpreted as descriptive, preliminary evidence supporting the generalizability of the archetype definitions, rather than as formal statistical confirmation of external validity; a quantitative assessment of cross-cohort stability represents an important direction for future work. Classification certainty in the external cohort was nonetheless high (mean APP = 0.965, ranging from 0.913 to 0.997 across archetypes), supporting confident and consistent assignment of external patients to the pre-established archetypes.

## Discussion

Patient classifications have been usually based on biological and molecular subtypes in breast cancer. The VBHC proposes to group patients on their characteristics and therapeutic pathway, which considerably influences the PCOs, the cornerstone of VBHC. This criterion of patient classification should help to identify response patterns and determine the most efficient therapeutic strategies (15, 16). In particular, benchmarking within a VBHC framework requires comparing outcomes among clinically and therapeutically comparable patients to avoid conflating case-mix differences with provider performance; without such classification, benchmarking risks biased comparisons and reduced actionability of results. This research aimed to overcome the limitation of the HCPC by using an alternative method to identify patient archetypes in breast cancer, and to design a patient classification tool to allocate new patients diagnosed with breast cancer into archetypes. Patient-centred outcomes were intentionally excluded from the LCA to avoid methodological circularity, as they constitute the outcomes later used for benchmarking within the VBHC framework. Previous work has shown that therapeutic pathways strongly influence these outcomes Gorostiza et al. (10), which reinforces the rationale for basing archetypes on treatment-related variables rather than on patient-reported measures. According to our results, the LCA provides an efficient and robust tool for defining and classifying patients diagnosed with breast cancer, also supported by classification performance checks and preliminary external cohort comparisons. However, this evidence of robustness and efficiency is preliminary and limited to the datasets and short follow-up analysed; further external validation and stability analyses are required before generalizing this claim. Therefore, LCA stability addresses the gap in classification of new patients into the defined archetypes.

Patients presenting DCIS were initially grouped in Arch-0 in accordance with the criteria set forth by clinical experts. The balance between adequate fit and moderate complexity across the eight models, estimated with their corresponding number of archetypes, indicated that the model providing six archetypes (Arch-1-6) was the most appropriate. It should be noted that although each archetype represents the patients who are most similar in therapeutic terms, not all patients share all the characteristics of the most representative patient of their archetype. While this could lead to inaccurate interpretations, the grouping of patients should yield significant overall results. We acknowledge that class membership is probabilistic and that individual variability within classes may limit the precision of class-level inferences for some subgroups.

Beyond retrieving the same number of archetypes as previous HCPC-based work (16), the clinical interpretation of the therapeutic pathways remains highly consistent across both methodologies. Major therapeutic trajectories—such as breast-conserving pathways with varying systemic regimens, mastectomy pathways stratified by reconstruction and axillary management, and high-intensity multimodality treatment—are clearly recognizable in both LCA and HCPC frameworks. This clinical concordance indicates that the underlying patient structure is robust and independent of the specific clustering technique. Crucially, LCA provides key methodological advantages over HCPC by replacing subjective tree-cutting decisions with a model-based probabilistic framework. This enables automated, uncertainty-aware classification of new patients using posterior probabilities, as confirmed by our Random Forest validation. While external validation showed consistent archetype distributions across centres, broader evaluation in diverse cohorts remains essential to confirm generalizability.

This allowed the LCA for the generation of fit statistics, which determine the most appropriate number of archetypes. This approach may be controversial since the fit statistics across M5-M8 have not definitively identified a single optimal fit. However, the balance between an adequate fit and parsimony led to the selection of the six-class model (M6) as the most appropriate. In addition to defining patient archetypes, the LCA has also been applied for classifying new patients into the defined archetypes. The LCA-derived classification tool demonstrated high concordance when evaluated against the Random Forest classifier. The results demonstrated that the LCA is a highly accurate tool that provides stability and robustness in the applied method. Consequently, the LCA is considered a more robust method than HCPC (20–22), addressing the gap in classification of new patients and allowing for the use of a single method to address two distinct purposes. Furthermore, the external validation showed similar patient archetypes in different centres, and consequently it might be scaled to other centres. In summary, the LCA provides an efficient and robust tool for defining and classifying patients diagnosed with breast cancer. We temper this conclusion by noting that external validation was performed in centres with organisational and population characteristics similar to the derivation cohort; broader validation in independent and diverse cohorts is needed to confirm transferability.

The implications of this study operate at three distinct levels. First, the methodological contribution lies in demonstrating that LCA can be used to derive statistically coherent and clinically interpretable patient archetypes, and in developing a classification tool capable of allocating new patients into these archetypes with high posterior probabilities. Second, the clinical usefulness of the archetypes relates to their potential to support pathway design and to identify groups of patients who may benefit from differentiated care strategies, although further work is required to assess their impact on clinical decisionmaking. Third, the applicability for benchmarking concerns the ability of these archetypes to enable fairer comparisons across providers by grouping patients with similar therapeutic pathways, thereby reducing case-mix heterogeneity. While these three dimensions are related, they are not equivalent, and the present study primarily provides evidence on the methodological dimension and its potential implications for clinical practice and benchmarking. We emphasise that, for benchmarking purposes, inadequate patient classification may conflate case-mix differences with provider performance, producing biased or misleading comparisons; thus, archetype-based grouping should be accompanied by sensitivity analyses and external validation before deployment in performance assessment. This study is not without limitations. The complete-case approach led to the exclusion of 684 patients (27.5% of the initial cohort) with missing age or surgery data in the administrative and clinical databases. These patients were older and had lower recorded treatment use than included patients (Supplementary Table S1), so missingness cannot be assumed to be random, and the archetypes may underrepresent older, less intensively treated patients. The archetypes should therefore be interpreted as describing patients with a fully documented therapeutic pathway. The short follow-up of 6-months in the context of breast cancer might not be considering the entire patient pathway for some patients, which might limit the usefulness of these results. The inherent imbalance in the distribution of patients across the various archetypes poses a significant challenge in terms of achieving precise classification. The classification algorithms have been trained with the response variable (*y/archetype*) estimated with LCA. As a result, the RF comparison should be interpreted as a test of robustness of the tool used to clasify new patients into the pre-established archetypes. Additional limitations include the need to assess class stability via resampling methods (e.g., bootstrap), evaluate sensitivity to alternative variable selections, and conduct further prospective and temporal validations in broader independent cohorts.

This paper shows that LCA provides a robust and transparent framework for identifying clinically meaningful patient archetypes in breast cancer and for developing a classification tool that can allocate new patients into these archetypes. The approach supports benchmarking within the VBHC framework by enabling fairer comparisons across providers based on groups of patients with similar therapeutic pathways. However, the evidence presented here is limited to a single clinical condition and to data from one healthcare system. Future research is needed to evaluate the external validity of the archetypes, to test the classification tool in independent cohorts, and to assess the transferability of this methodological framework to other medical conditions and organisational contexts. Longer follow-up periods would allow the assessment of how patient archetypes evolve over time and whether transitions between archetypes are clinically meaningful. In addition, archetypes may require periodic updating to reflect changes in therapeutic pathways and emerging clinical practices.

## Data Availability

The data underlying this article cannot be shared publicly due to privacy and ethical considerations related to the individuals who participated in the study. Anonymized data are available from the corresponding author upon reasonable request.
