# Supplementary material for: From therapeutic pathways to patient archetypes: a latent class analysis framework for Value-Based Healthcare benchmarking
Source: Front Health Serv. 2026 Oct 21;6:1789729. doi: 10.3389/frhs.2026.1789729 (PMC13731109; doi:10.3389/frhs.2026.1789729)
Supplement: Supplementary file 1 [file Supplementaryfile1.pdf]

Table S1. Comparison of patients included and excluded from the latent class analysis

	**Included (N=1,801)**	**Excluded (N=684)**	**p-value**
**PATIENT CHARACTERISTICS**			
Age at diagnosis			<0.001
(0,50]	457 (25.37%)	58 (8.48%)	
(50,70]	983 (54.58%)	123 (17.98%)	
(70,97]	361 (20.04%)	116 (16.96%)	
NA (missing)	0 (0%)	387 (56.58%)	
**TUMOUR CHARACTERISTICS**			
Tumour type			0.895[a]
Ductal carcinoma in situ	231 (13.71%)	32 (4.68%)	
Invasive ductal carcinoma	1210 (67.18%)	141 (20.61%)	
Invasive lobular carcinoma	156 (8.66%)	18 (2.63%)	
Other carcinoma	84 (4.66%)	8 (1.17%)	
NA (missing)	104 (5.77%)	485 (70.91%)	
**TREATMENT CHARACTERISTICS**			
Surgery			<0.001[b]
BCT	1353 (75.13%)	87 (12.72%)	
Mast. w/o im.rec.	181 (10.05%)	7 (1.02%)	
Mast. with im.rec.	267 (14.82%)	90 (13.16%)	
NA (missing)	0 (0%)	500 (73.10%)	
Surgery to axilla			<0.001[b]
None	215 (11.94%)	532 (77.78%)	
Sentinel lymph node biopsy	1291 (71.68%)	116 (16.96%)	
Axillary clearance	288 (15.99%)	33 (4.82%)	
Axillary sampling	7 (0.39%)	1 (0.15%)	
NA (missing)	0 (0%)	2 (0.29%)	
Chemotherapy			<0.001
No	1485 (82.5%)	654 (95.6%)	

Yes	316 (17.5%)	30 (4.4%)	
Radiotherapy			<0.001
No	874 (48.5%)	600 (87.7%)	
Yes	927 (51.5%)	84 (12.3%)	
Hormonal therapy			<0.001
No	886 (49.2%)	618 (90.4%)	
Yes	915 (50.8%)	66 (9.6%)	

Percentages are calculated within each column (Included, N=1,801; Excluded, N=684).

P-values calculated using chi-squared test, or Fisher's test

[a] This p-value reflects only patients with known tumour type; missingness was far more common among excluded patients (70.9%) than included patients (5.8%), which the test does not capture.

[b] Surgery and Surgery to axilla are among the variables used to define inclusion in the latent class analysis;

Table S2. Confusion matrix for Class prediction

	**LCA**					
**RF**	**Arch-1**	**Arch-2**	**Arch-3**	**Arch-4**	**Arch-5**	**Arch-6**
**Arch-1**	40	0	0	0	0	0
**Arch-2**	0	33	0	0	0	0
**Arch-3**	0	0	102	0	0	0
**Arch-4**	1	0	0	18	0	0
**Arch-5**	0	0	0	0	70	0
**Arch-6**	0	0	0	0	0	50

Table S3. Performance metrics from the comparison of LCA vs RF classification

Archetype	Sensitivity (%)	Specificity (%)	Accuracy (%)
**Arch-1**	97.5	100	98.75
**Arch-2**	100	100	100
**Arch-3**	100	100	100
**Arch-4**	100	99.6	99.65
**Arch-5**	100	100	100
**Arch-6**	100	100	100

SD: standard deviation

Table S4. Description of archetypes with LCA prediction –remaining Integrated Health Organizations

**LCA (Prediction)**	Arch-0	**Arch-1** (N=48)	**Arch-2** (N=123)	**Arch-3** (N=111)	**Arch-4** (N=8)	**Arch-5** (N=15)	**Arch-6** (N=47)
**Age at diagnosis (cat):**							
<50	13 (21.7%)	25 (52.1%)	38 (30.9%)	21 (18.9%)	6 (75.0%)	2 (13.3%)	17 (36.2%)
(50,70]	35 (58.3%)	20 (41.7%)	56 (45.5%)	48 (43.2%)	2 (25.0%)	7 (46.7%)	23 (48.9%)
>70	10 (16.7%)	3 (6.3%)	29 (23.6%)	42 (37.8%)	0 (0%)	6 (40.0%)	7 (14.9%)
**Surgery:**							
BCS	41 (68.3%)	0 (0%)	80 (65.0%)	111 (100%)	0 (0%)	14 (93.3%)	47 (100%)
MWoR	7 (11.7%)	0 (0%)	43 (35.0%)	0 (0%)	5 (62.5%)	0 (0%)	0 (0%)
MWR	10 (16.7%)	48 (100%)	0 (0%)	0 (0%)	3 (37.5%)	1 (6.7%)	0 (0%)
**Surgery to axilla:**							
None	29 (48.3%)	0 (0%)	2 (1.6%)	7 (6.3%)	1 (12.5%)	1 (6.7%)	0 (0%)
SLNB	10 (16.7%)	24 (50.0%)	91 (73.9%)	0 (0%)	0 (0%)	0 (0%)	0 (0%)
Axillary clearance	2 (3.3%)	16 (33.3%)	22 (17.9%)	32 (28.8%)	0 (0%)	11 (73.3%)	0 (0%)
Axillary sampling	14 (23.3%)	8 (16.7%)	8 (6.5%)	72 (64.9%)	7 (87.5%)	3 (20.0%)	47 (100%)
**Chemotherapy YES**	2 (3.3%)	27 (56.3%)	40 (32.5%)	21 (18.9%)	8 (100%)	11 (73.3%)	47 (100%)
**Radiotherapy YES**	37 (61.7%)	20 (41.7%)	96 (78.0%)	102 (91.9%)	8 (100%)	13 (86.7%)	45 (95.7%)
**Hormonal therapy YES**	22 (36.7%)	40 (83.3%)	110 (89.4%)	111 (100%)	8 (100%)	1 (6.7%)	33 (70.2%)

LCA: Latent Class Analysis
BCS: Breast Conserving Surgery
MWR: Mastectomy with immediate reconstruction
MWoR: Mastectomy without immediate reconstruction
SNLB: Sentinel lymph node biopsy
